# Expanding the Zebrafish Genetic Code through Site-Specific Introduction of Azido-lysine, Bicyclononyne-lysine, and Diazirine-lysine

**DOI:** 10.3390/ijms20102577

**Published:** 2019-05-26

**Authors:** Junetha Syed, Saravanan Palani, Scott T. Clarke, Zainab Asad, Andrew R. Bottrill, Alexandra M.E. Jones, Karuna Sampath, Mohan K. Balasubramanian

**Affiliations:** 1 Centre for Mechanochemical Cell Biology and Division of Biomedical Sciences, Warwick Medical School, University of Warwick, Gibbet Hill Road, CV4 7AL Coventry, UK; junetha.syedjabarulla@oncology.ox.ac.uk (J.S.); s.palani@warwick.ac.uk (S.P.); S.T.Clarke@warwick.ac.uk (S.T.C.); z.asad@warwick.ac.uk (Z.A.); k.sampath@warwick.ac.uk (K.S.); 2 Proteomics Research Technology Platform and School of Life Sciences, University of Warwick, Gibbet Hill Road, CV4 7AL Coventry, UK; andrew.bottrill@warwick.ac.uk (A.R.B.); alex.jones@warwick.ac.uk (A.M.E.J.)

**Keywords:** genetic code expansion, zebrafish, unnatural amino acid, protein engineering, proteomics, crosslinking, click chemistry

## Abstract

Site-specific incorporation of un-natural amino acids (UNAA) is a powerful approach to engineer and understand protein function. Site-specific incorporation of UNAAs is achieved through repurposing the amber codon (UAG) as a sense codon for the UNAA, using a tRNA^CUA^ that base pairs with an UAG codon in the mRNA and an orthogonal amino-acyl tRNA synthetase (aaRS) that charges the tRNA^CUA^ with the UNAA. Here, we report an expansion of the zebrafish genetic code to incorporate the UNAAs, azido-lysine (AzK), bicyclononyne-lysine (BCNK), and diazirine-lysine (AbK) into green fluorescent protein (GFP) and glutathione-s-transferase (GST). We also present proteomic evidence for UNAA incorporation into GFP. Our work sets the stage for the use of AzK, BCNK, and AbK introduction into proteins as a means to investigate and engineer their function in zebrafish.

## 1. Introduction

Site-directed mutagenesis is a powerful approach to engineer and investigate protein function. In site-directed mutagenesis, one or more amino-acids in a protein are replaced with other canonical amino acid(s), often with properties in stark contrast with the original amino-acid (canonical is defined to encompass the 20 amino acids found in proteins) [1,2]. The replacement of native amino-acids within a protein with other amino-acids allows the functional dissection of individual amino-acid residues as well as domains in proteins. The ability to introduce unnatural amino-acids (UNAAs) with novel properties in a site-specific manner is an important extension to site-directed mutagenesis and has the potential to revolutionize the investigation and engineering of protein function [3,4,5,6]. The site-specific incorporation of UNAAs has been made possible through the availability of UNAAs with a plethora of useful properties, combined with the availability of repurposed codons and orthogonal tRNA and aminoacyl tRNA synthetases (aaRS) for these amino acids [7,8,9]. A large number of UNAAs are available that permit, among others, photo-crosslinking, photo-uncaging, and click chemistry.

The zebrafish is an attractive vertebrate model organism to uncover mechanisms of development and disease [10]. We set out to establish genetic code expansion strategies in zebrafish to facilitate structural and functional studies of proteins. Previous work has shown that azido-phenylalanine [6] and some lysine derivatives [11] can be introduced into zebrafish proteins through genetic code expansion. We sought to incorporate three useful amino-acids—azido-lysine (AzK), bicyclononyne-lysine (BCNK), and diazirine-lysine (AbK)—which to our knowledge had not been previously incorporated into proteins in zebrafish. We show the successful incorporation of these amino-acids into translation products of synthetic green fluorescent protein (GFP) and glutathione-S-transferase (GST) mRNAs injected into zebrafish embryos.

## 2. Results and Discussion

**Figure 1:**
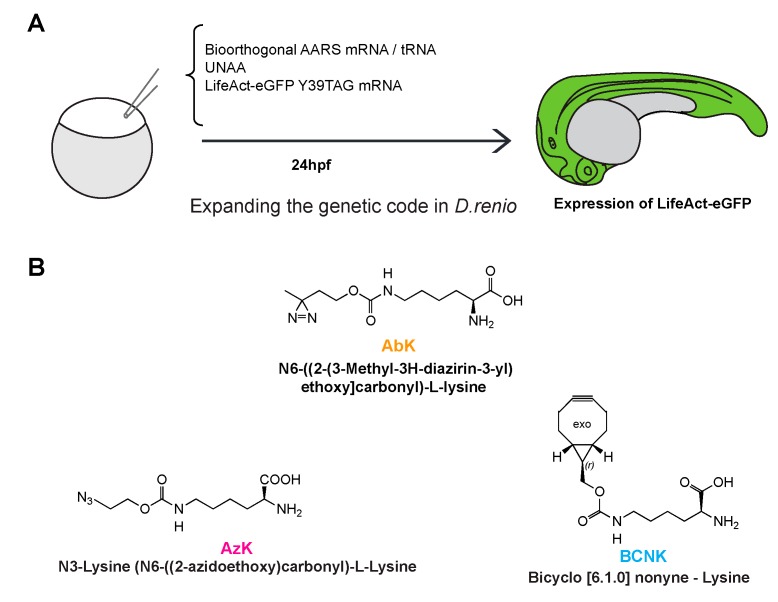
(**A**). The strategy for genetic code expansion in zebrafish embryos. (**B**). The structures of the unnatural amino-acids (azido-lysine, bicyclononyne-lysine, and diazirine-lysine) used in this study.

The strategy we employed to expand the genetic code in zebrafish embryos is shown in Figure 1A. Briefly, 1–2 cell zebrafish embryos were injected with a mixture carrying in vitro transcribed tRNA^CUA^ and mRNA for the orthogonal aaRS, an amber (UAG) codon containing mRNA for the target protein (GFP or GST), and an UNAA. The tRNA and mRNA for the aaRS are collectively referred to as the UNAA cocktail, which was used with or without the UNAA as indicated in the appropriate experiments performed. GFP sequences were fused with LifeAct, a short peptide that binds to the F-actin cytoskeleton [12], so as to distinguish UNAA-protein expression from background autofluorescence, which might confound the evaluation of successful genetic code expansion. Deiters and colleagues have successfully incorporated a variety of lysine derivatives, including a photo-cleavable amino acid, into zebrafish proteins [11]. In this work, we tested other lysine derivatives not used previously in zebrafish to further expand the tool kit for use in zebrafish. AbK and AzK were chosen for their ability to generate reactive carbene and nitrene, respectively, upon exposure to ultraviolet light. The carbene and nitrene groups cause covalent cross-links with amino-acids in close proximity (~3–12 Å), allowing for the investigation of direct binding partners [13,14]. Azido-lysine can also be used in click chemistry reactions (strain-promoted azide-alkyne cycloaddition-SPAAC). BCNK can be used in different kinds of click chemistry reactions (strain-promoted inverse electron demand Diels-Alder cycloaddition-SPIDEAC) [15]. We used two different tRNA synthetases: 1) *Methanosarcina mazei* PylRS (carrying the amino acid substitutions Y360A and Y384F) for incorporating AzK and BCNK [16] and 2) *Methanosarcina barkeri PylRS* (L274M, C313A, Y349F) for integrating AbK [17]. In all cases, *Methanosarcina* pyrrolysine tRNA^Pyl-CUA^ that base-pairs with UAG codons was introduced into the injected mRNA and was charged by either aaRS that was used [18]. All injected GFP and GST mRNAs contained an ochre UAA codon for translation termination.

**Figure 2:**
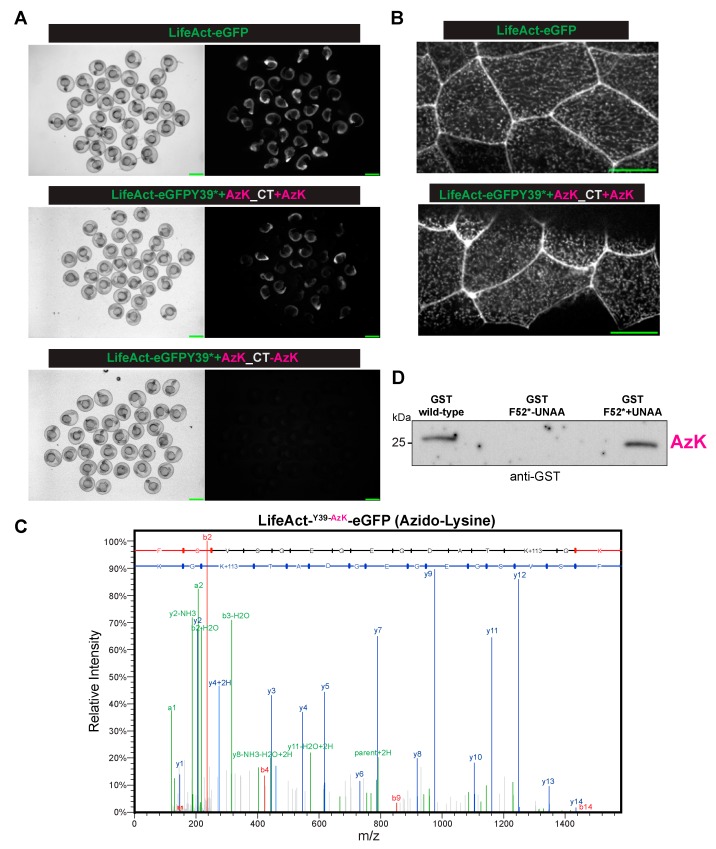
The site-specific incorporation of azido-lysine in the zebrafish embryos. (**A**) The Brightfield (left) or fluorescent (right) images of the zebrafish embryos injected with mRNA(s) for LifeAct-eGFP (top panel) or LifeAct-eGFP Y39* (codon for Y39 converted to stop codon UAG) with the orthogonal tRNA^Pyl CUA^-mRNA aaRS for AzK/BCNK, with (middle panel) AzK but not without (bottom panel) AzK. Scale bar, 1 mm (**B**). The localization of LifeAct-eGFP or LifeAct-eGFP Y39AzK to cell–cell junctions and cortical speckles in the dechorionated embryos injected as described in (A). Scale bar represents 20 µm. (**C**) The mass-spectrometry identified AzK incorporation in LifeAct-eGFP Y39AzK. The lysates were prepared from ~1000 embryos injected with mRNA for LifeAct-eGFP Y39* with the orthogonal tRNA^Pyl-CUA^ -mRNA aaRS for AzK/BCNK with AzK. LifeAct-eGFP Y39AzK was immunoaffinity purified using GFP nanobodies and processed for mass spectrometry as described in the methods section. The MS/MS spectrum of precursor *m*/*z* = 791.3606 (M+2) corresponded to tryptic peptide FSVSGEGEGDATKGK with azido-lysine incorporated at the indicated position. The full y-ion fragment series is highlighted in blue. (**D**) An immunoprecipitation-Western blot from the zebrafish embryos injected with mRNA(s) for GST (left lane) or GST F52* (codon for F52 converted to stop codon UAG) with the orthogonal tRNA^Pyl-CUA^ -aaRS pair for AzK/BCNK, without (middle lane) or with (right lane) AzK. The immunoprecipitations and Western blots were performed with antibodies against GST.

In the embryos injected with LifeAct-eGFP mRNA, robust GFP expression was observed, and at a subcellular level, and as expected, the majority of LifeAct-eGFP localized to cell–cell junctions and in cortical speckles (Figure 2A,B). In the control embryos, mRNA encoding LifeAct-eGFP Y39* (where a UAG codon replaced the codon for Y39) was injected together with the appropriate AzK/BCNK cocktail, but without the UNAA, no GFP signal was detected (Figure 2A). By contrast, when the AzK/BCNK cocktail was injected along with AzK, strong GFP expression was detected (Figure 2A). At the subcellular level, LifeAct-eGFP Y39AzK localized to cell–cell junctions and to cortical speckles, similar to LifeAct fused to wild-type eGFP. The fact that GFP expression was detected only in the presence of AzK strongly suggested the specificity of the AzK/BCNK cocktail in zebrafish embryos. To further validate the site-specific incorporation of AzK into LifeAct-eGFP at position Y39, LifeAct-eGFP was immunoprecipitated using GFP-Trap and analysed by nano-LC-ESI-MS/MS after digestion with trypsin. Proteomic studies confirmed this suggestion. The LifeAct-eGFP peptide (FSVSGEGEGDATKGK) that incorporated azido-lysine at position 39 with a 113 Da adduct (the predicted mass of the added azido-moiety), was readily identified by mass spectrometry as a M+2 ion of 791.3606 *m*/*z*. We also successfully incorporated AzK into GST and detected GST through immunoprecipitation followed by western blotting in embryos injected with GSTF52* and the AzK/BCNK cocktail with AzK but not when AzK was not included (Figure 2D). Collectively, these experiments established that we had successfully expanded the genetic code of zebrafish to site-specifically introduce AzK.

**Figure 3:**
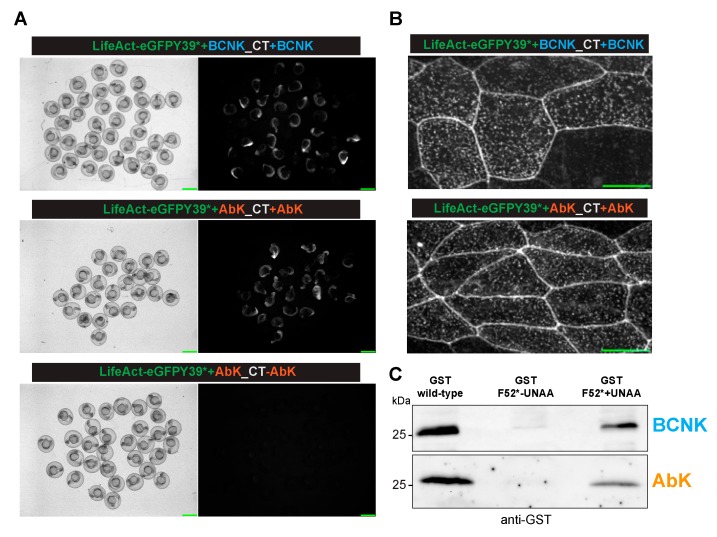
The site-specific incorporation of bicylononyne-lysine and diazirine-lysine in the zebrafish embryos. (**A**) The Brightfield (left) or fluorescent (right) images of the zebrafish embryos injected with mRNA(s) for LifeAct-eGFP Y39* with the orthogonal tRNA^Pyl-CUA^ -mRNA aaRS for AzK/BCNK with (top panel) BCNK. Shown also are fields with multiple examples of each of the zebrafish embryos injected with mRNA(s) for LifeAct-eGFP Y39* with the orthogonal tRNA^Pyl-CUA^ -mRNA aaRS for AbK, with (middle panel) or without (bottom panel) AbK. (**B**) The localization of LifeAct-eGFP Y39BCNK (top panel) and LifeAct-eGFP Y39AbK to cell-cell junctions and cortical speckles in the dechorionized embryos injected as described in (A). (**C**) Shown in the top panel is an IP-Western blot from the zebrafish embryos injected with mRNA(s) for GST (left lane) or GFT F52* with the orthogonal tRNA^Pyl-CUA^ -aaRS pair for AzK/BCNK, without (middle lane) or with (right lane) BCNK. The bottom panel shows a similar experiment showing the incorporation of AbK into the zebrafish embryos using the appropriate amino-acid and the tRNA^Pyl-CUA^ –aaRS cocktail for AbK. The immunoprecipitations and Western blots were performed with antibodies against GST.

We next tested for the incorporation of BCNK into the proteins in the zebrafish embryos using the strategy employed in Figure 2, except that BCNK was used instead of AzK since the same aaRS was capable of charging the tRNA with AzK and BCNK. We found GFP expression when the embryos were injected with BCNK (Figure 3A; BCNK panel, and see Figure 3B for localization to cell–cell junctions and cortical speckles) but not in the absence of BCNK (Figure 2A). Similarly, GST expression could be readily detected when the mRNA for GST F52* was co-injected with the AzK/BCNK cocktail and BCNK (Figure 3C). We detected a faint band in the sample that was not injected with BCNK. It is possible that there may have been a very low level/inefficient charging of tRNAPyl-CUA with a cellular aaRS or an inefficient binding of a natural amino acid to aaRS AzK/BCNK. Finally, we tested if AbK could be introduced into proteins in zebrafish through genetic code expansion. To this end, we injected the zebrafish embryos with the AbK cocktail with or without AbK. In these experiments, we found that GFP expression and fluorescence (Figure 3A,B) and GST expression were detected only when the embryos were injected with AbK (Figure 3C).

In summary, we present evidence for the site-specific incorporation of three unnatural amino acids through genetic code expansion into two different reporter proteins in zebrafish embryos. These amino-acids can be used in photo-crosslinking and/or click chemistry experiments. While we have clearly demonstrated the incorporation of an UNAA into GFP through proteomics experiments, further developments are needed to make this method useful in a large number of biological studies. First, we were unable to carry out photo-crosslinking under conditions in which we successfully carried out photo-crosslinking in bacterial, yeast, and mammalian cells. Second, the UNAAs need to be injected into embryos, since in our conditions, even after the removal of the chorion, they did not pass cell membranes. It is noteworthy that Deiters and colleagues also injected lysine-derived UNAAs into zebrafish embryos [11]. Therefore, conditions that facilitate the membrane permeability of UNAAs into zebrafish embryos need to be investigated. Finally, transgenic lines expressing the orthogonal AzK/BCNK and Abk tRNA-aaRS pairs need to be created, such that this powerful technology can be used in free-feeding adult fish. Notwithstanding these limitations, the methods we report provide a platform for the further development of this powerful technology to enable the precise engineering of proteins in zebrafish embryos. In addition to the traditional uses of this system, the fact that the expression of marker proteins is only achieved in the presence of the UNAA suggests that the method we describe can also be used for the inducible expression of proteins in zebrafish.

## 3. Materials and Methods

### 3.1. Un-Natural Amino-Acids (UNAAs)

AzK (N3-lysine (N6-((2-azidoethoxy) carbonyl)-l-lysine, SC-8027) and Exo-BCNK (bicyclo [6.1.0] nonyne–lysine, SC-8016) were purchased from Sirius fine chemicals SiChem GmbH. AbK (N6-[[2-(3-Methyl-3H-diazirin-3-yl) ethoxy] carbonyl]-l-lysine, 5113) was purchased from Tocris. 200 mM stock solutions of each UNAA were prepared by dissolving them in 85% 0.2 M NaOH and 15% DMSO.

### 3.2. Plasmid Construction

The two different tRNA synthetases—1) the double mutant of wild-type *Methanosarcina mazei* PylRS (Y360A and Y384F) for incorporating AzK and BCNK and 2) the mutant *Methanosarcina barkeri PylRS* (L274M, C313A, Y349F) for integrating AbK—were cloned into the pCS2+ vector. Wild-type LifeAct-eGFP and GST were cloned into the pCS2+ vector. The TAG codons were introduced at the Y39 position of eGFP and at the Y52 position of GST, respectively. All cloning procedures were performed using the in-fusion cloning kit (Clontech).

### 3.3. In Vitro Transcription

The coding sequences cloned into the pCS2+ vector was linearized by NotI digestion. 500 ng of the linearized product was used as a template in a 20 μL reaction to synthesize the corresponding mRNA using a mMESSAGE mMACHINE SP6 Transcription Kit (AM1340), which was further purified by phenol:choloform:isoamyl alcohol extraction.

The DNA templates for synthesizing amber suppressor pyrrolysine tRNA (PylT) of the Methonosarcina species were ordered from sigma as oligonucleotides preceded by the T7 promoter.

The sequences of the templates were as follows. PylT forward: 5′-ATTCGTAATACGACTCACTATAGGAAACCTGATCATGTAGATCGAATGGACTCTAAATCCGTTCAGCCGGGTTAGATTCCCGGGGTTTCCGCCA-3′. PylT Reverse: 5′-TGGCGGAAACCCCGGGAATCTAACCCGGCTGAACGGATTTAGAGTCCATTCGATCTACATGATCAGGTTTCCTATAGTGAGTCGTATTACGAAT-3′.

The above-mentioned complementary oligonucleotides were annealed (annealing buffer: 10 mM Tris pH 8.0, 50 mM NaCl, 1 mM EDTA), and 1 μg of the annealed oligonucleotides were used as templates for in vitro transcription following the mMESSAGE mMACHINE™ T7 Transcription Kit protocol.

### 3.4. Microinjection of Zebrafish Embryos

Wild-type embryos were obtained by natural mating using standard procedures in accordance with institutional animal care regulations at the University of Warwick. One-cell stage zebrafish embryos were injected with 2 nL from the total 1.5 μL injection mixture (0.25 μL of 200 ng/μL LifeAct-eGFP/GST mRNA, 0.75 μL of 200 ng/μL tRNA synthetase mRNA, 0.25 μL of 4000 ng/μL of tRNA, 0.25 μL of 100 mM UNAA). The injected embryos were collected 24 h after fertilization for biochemical/cell biological analyses.

### 3.5. Imaging

The embryos were incubated at 28.0 °C overnight after injection. Clutches of the chorionated embryos were imaged at approximately 24 h post-fertilisation on plastic petri dishes using a SMZ18 stereo microscope equipped with a Teledyne Phometrics CoolSnap HQ2 CCD camera. A Nikon P2-SHR Plan Apo 1×/0.15 NA objective lens was used to obtain a final pixel size of 8.85 μm/pixel. A Nikon Intenslight C-HGFI illuminator was used for excitation. Image acquisition was automated using Fiji micro-manager [19]. The embryos were thence dechorionated and embedded in 0.7% low melting agarose on No. 0 coverslips (MatTek 35 mm uncoated dishes). The dorsal anterior was imaged at approximately 24 h post-fertilisation using an Andor Revolution XD confocal system, assembled on a Nikon Eclipse Ti inverted microscope with a spinning disc confocal Yokogawa CSU-X1 unit and an Andor iXon Ultra EMCCD camera. A Nikon Fluor 40×/1.30 NA objective lens was used to obtain a final pixel size of 0.2 μm/pixel. All images were acquired with a Z-step size of 0.5 μm. A 561 nm laser line was used for excitation. Image acquisition was automated using Andor IQ3 software. A maximum intensity projection of the optical stack was performed using Fiji [20].

### 3.6. Western Blotting and Immunoprecipitation

The one-cell stage zebrafish embryos were injected with the appropriate cocktail (Orthogonal tRNA synthetase, tRNA and GST^F52^TAG or LifeAct^Y39^TAG-eGFP RNA) and UNAAs (AzK, AbK and BCNK). The embryos were collected after 24 h, and the lysates were prepared using a standard RIPA buffer (50 mM Tris (pH 8.0); 150 mM NaCl; 1% NP-40; 0.5% deoxycholate; 0.1% SDS and 1× cocktail protease inhibitors), incubated with the buffer on ice for 15 min, and further clarified at 14,000 RPM for 15–20 min at 4 °C. The clarified lysates were heated for 5 min at 95 °C with a 4× sample loading buffer loaded on 12% SDS-PAGE gels, and transferred to a nitrocellulose membrane (GE Healthcare, Chicago, USA), and immunoblotting was performed using anti-GFP-HRP (sc-9996 HRP) and anti-GST-HRP (sc-138 HRP).

The lysates from the embryos (>800) were prepared as mentioned above for the immunoprecipitation experiments. The clarified lysates were incubated with a pre-washed GFP-Trap (chromotek) in a RIPA buffer for 12–16 hr at 4 °C. GFP-Trap beads were washed with a RIPA buffer 5–6 times prior to loading onto 12% SDS-PAGE gels for mass spectrometry.

### 3.7. Sample Preparation

Samples from the zebrafish embryo extracts were run in 12% SDS-PAGE mini-gels until the dye front was 1 cm from the bottom. The gels were washed with deionised water three times (5 min each) and stained with Coomassie blue (SimplyBlueStain, Invitrogen) overnight and de-stained with deionised water for 4–6 h. A LifeAct-eGFP-carrying unnatural amino acid (AzK) band (around 27–29 KDa) in the gel was cut into 1 mm^3^ pieces for in-gel tryptic digestion [21] prior to MS analysis.

### 3.8. NanoLC-ESI-MS/MS Analysis and UNAA Identification

Reversed phase chromatography was used to separate tryptic peptides prior to mass spectrometric analysis. Two columns were utilised: An Acclaim PepMap µ-precolumn cartridge 300 µm i.d. × 5 mm 5 μm 100 Å and an Acclaim PepMap RSLC 75 µm × 25 cm 2 µm 100 Å (Thermo Scientific). The columns were installed on an Ultimate 3000 RSLCnano system (Dionex). Mobile phase buffer A was composed of 0.1% formic acid in water and mobile phase B 0.1% formic acid in acetonitrile. The samples were loaded onto the µ-precolumn equilibrated in 2% aqueous acetonitrile containing 0.1% trifluoroacetic acid for 5 min at 10 µL min-1 after which peptides were eluted onto the analytical column at 250 nL min-1 by increasing the mobile phase B concentration from 4% B to 25% over 37 min, then to 35% B over 10 min, and to 90% B over 3 min, followed by a 10 min re-equilibration at 4% B [22].

The eluting peptides were converted to gas-phase ions by means of electrospray ionization and were analysed on a Thermo Orbitrap Fusion (Thermo Scientific). Survey scans of peptide precursors from 375 to 1575 *m*/*z* were performed at 120K resolution (at 200 *m*/*z*) with a 2 × 105 ion count target. Tandem MS was performed by isolation at 1.2 Th using the quadrupole, HCD fragmentation with normalized collision energy of 33, and rapid scan MS analysis in the ion trap. The MS2 ion count target was set to 1× 104, and the max injection time was 200 ms. Precursors with charge state 2–6 were selected and sampled for MS2. The dynamic exclusion duration was set to 25 s with a 10-ppm tolerance around the selected precursor and its isotopes. Monoisotopic precursor selection was turned on, and the instrument was run on top speed mode for 2 s.

### 3.9. Data Analysis

The raw data were searched using MaxQuant [23] (version 1.6.2.6) against the sequence of the construct, Uniprot Danio rerio reference proteome (www.uniprot.org/proteomes/UP000000437; 20 March 2019) and the MaxQuant common contaminant database. For the database search, peptides were generated from a tryptic digestion with up to two missed cleavages and the carbamidomethylation of cysteines as fixed modifications, and the variable modifications used were for the oxidation of methionine, acetylation of the protein N-terminus, azido-lysine (+C3H3O2N3).
